# CAPZA1 deficiency disrupts sperm flagellar structure and motility, potentially involving the p300/SLC7A11 pathway

**DOI:** 10.3389/fendo.2026.1744836

**Published:** 2026-03-04

**Authors:** Hui Lu, Guoxuan Li, Jiajia Hu, Hailing Ruan, Liqiang Zhao, Yejuan Li, Anguo Wang

**Affiliations:** 1 Reproductive Medical Center, Hainan Women and Children’s Medical Center, Haikou, Hainan, China; 2 Hainan Medical University, The University of Hong Kong Joint Laboratory of Tropical Infectious Diseases, Key Laboratory of Tropical Translational Medicine of Ministry of Education, School of Basic Medicine and Life Sciences, Hainan Medical University, Haikou, Hainan, China

**Keywords:** asthenozoospermia, CAPZA1, dynein arms, fibrous sheath, p300/SLC7A11 pathway, sperm motility

## Abstract

**Objective:**

To investigate the genetic and molecular role of CAPZA1 in asthenozoospermia and its impact on sperm motility and flagellar integrity.

**Methods:**

Whole-exome sequencing (WES) was first performed in an infertile family with asthenozoospermia to identify candidate variants. The CAPZA1 variant was further screened by Sanger sequencing in 20 infertile men with asthenozoospermia and 20 age-matched fertile controls. CAPZA1 expression and sperm motility parameters were assessed by Western blot and computer-assisted semen analysis, respectively. Structural abnormalities were examined using transmission electron microscopy (TEM). *In vitro* CAPZA1 knockout (KO-CAPZA1) was achieved in isolated mouse round spermatids using CRISPR-Cas9, followed by RT-qPCR, Western blot, ELISA for cystine levels, and thiol quantification to assess downstream effects. Protein localization of DNAH9 and FSCN1 was analyzed by immunofluorescence. *In vivo* CAPZA1 deletion was induced via adeno-associated virus (AAV)-mediated CRISPR-Cas9 delivery into mouse testes, and subsequent sperm motility, protein expression, and ultrastructure were evaluated.

**Results:**

A rare homozygous missense mutation in CAPZA1 (c.11T>C, p.Phe4Ser) was first identified by WES in the proband of an infertile family and was subsequently detected by Sanger sequencing in 3 of 20 asthenozoospermic patients. CAPZA1 protein expression was significantly reduced in mutant sperm, with a strong positive correlation to progressive motility (*r* = 0.849, *p* < 0.001). TEM revealed disorganized flagellar ultrastructure, including asymmetric fibrous sheath and partial dynein arm loss. In KO-CAPZA1 mouse spermatids, p300/CBP, SLC7A11, H3K27ac expression were decreased. Reduced cystine content and increased DTNB-reactive thiol groups after TCEP reduction indicated disrupted thiol/disulfide homeostasis. DNAH9 and FSCN1 expression and localization were disrupted in KO-CAPZA1 cells. KO-CAPZA1 in mice resulted in significantly decreased sperm progressive motility (*p* < 0.001) and abnormal axonemal structure, without affecting testicular morphology or sperm count.

**Conclusion:**

CAPZA1 deficiency impairs sperm motility and flagellar architecture through disrupted cytoskeletal protein regulation and redox imbalance, and represents a novel genetic contributor to asthenozoospermia.

## Introduction

Male infertility is a leading cause of reproductive failure and has become a critical global issue in male reproductive health (1). The primary etiology involves impaired sperm quality, which presents clinically as azoospermia, oligozoospermia, asthenozoospermia, and teratozoospermia (2). Among these, asthenozoospermia is the most common form of male infertility, accounting for approximately 20%-40% of cases (3). According to the World Health Organization (WHO) criteria, asthenozoospermia is diagnosed when progressive motility is less than 32% with otherwise normal semen parameters (4). The causes of asthenozoospermia are diverse, including genetic mutations, reproductive tract infections, varicocele, and endocrine disorders (5, 6). However, the etiology remains unclear in 30%-40% of patients (7). In recent years, increased psychological stress, environmental pollution, and the spread of sexually transmitted infections may have contributed to the rising incidence of asthenozoospermia (5). The relaxation of fertility policies and the acceleration of population aging further pose significant challenges to fertility rates.

Genetic mutations have been recognized as important contributors to asthenozoospermia. The structural integrity of the sperm flagellum is essential for motility, and defects in the flagellum are a major cause of impaired sperm motility (8). Mutations in genes encoding flagellar components, such as DNAH7 (9) and CFAP47 (10), have been associated with reduced sperm motility. Moreover, defects in dynein genes can result in the loss of dynein motor function, leading to impaired flagellar beating (11). For instance, loss-of-function mutations in DNALI1 disrupt the assembly of inner dynein arms and fibrous sheaths, ultimately leading to asthenozoospermia (12). Capping actin protein of muscle Z-line subunit alpha 1 (CAPZA1) is a regulatory protein that binds to the barbed (+) end of actin filaments to inhibit elongation, thereby participating in filament capping, stabilization, and disassembly, and regulating cytoskeletal dynamics, cellular motility, morphology, and signal transduction (13). Arafat M et al. demonstrated that a recessive mutation in the actin-associated protein CATIP may contribute to asthenozoospermia by disrupting actin polymerization and cytoskeletal regulation (14). A recent study also reported CAPZA1 downregulation in microarray analyses of spermatogenic cells from three patients with non-obstructive azoospermia (15). Based on these findings, this study further investigates the potential role and underlying mechanism of CAPZA1 in asthenozoospermia.

Additionally, lysine acetylation is a common post-translational modification of histones that plays a key role in chromatin remodeling and gene regulation (16). p300/CBP is a key histone acetyltransferase responsible for acetylation of histone H3 at lysine 27 (H3K27ac), facilitating chromatin accessibility and transcriptional activation (17). Previous studies by Tsugawa et al. reported a regulatory link between histone H3 acetylation and CAPZA1 expression, showing that increased acetylation at the CAPZA1 promoter upregulates its transcription under oxidative stress (18). While this establishes that CAPZA1 is a target of epigenetic regulation, it remains unclear whether CAPZA1 itself can exert feedback modulation on chromatin modifiers. Here, we hypothesize that CAPZA1 deficiency may, in turn, impair the activity of histone acetyltransferases like p300/CBP, leading to reduced H3K27ac levels and altered gene expression.

Solute carrier family 7 member 11 (SLC7A11) is a critical amino acid transporter mediating the exchange of extracellular cystine and intracellular glutamate (19). In spermatozoa, pharmacological inhibition of the cystine/glutamate antiporter SLC7A11 reduces intracellular glutathione and mitochondrial activity, supporting a role for SLC7A11-mediated cystine uptake in maintaining sperm redox homeostasis (20). Although disulfide bonds are known to contribute to the structural integrity of flagellar components, their precise role in the assembly of inner dynein arms and fibrous sheaths remains poorly understood. Notably, the absence of dynein arm and fibrous sheath proteins has been strongly associated with asthenozoospermia (21), yet the regulatory mechanisms governing their assembly are still unclear. This study aims to investigate the core mechanisms of sperm flagellar dysfunction by focusing on CAPZA1, SLC7A11, and their associated molecular pathways. We seek to elucidate the pathogenesis of asthenozoospermia and identify potential therapeutic targets.

## Materials and methods

### Study subjects

This study enrolled 20 infertile male patients between February 20, 2025 and June 10, 2025, as well as 20 age-matched fertile men as healthy controls. All participants were recruited according to the WHO 2010 criteria, and classified based on progressive motility into the asthenozoospermia or control group (22). The study protocol was approved by the Ethics Committee of Hainan Medical University (Approval No. 2025-IRB-455), and written informed consent was obtained from all subjects in accordance with the Declaration of Helsinki. Eligible participants were men aged 18–45 years, with at least two semen analyses showing progressive motility < 32% and total motility < 40%, normal reproductive hormone levels (including follicle-stimulating hormone, testosterone, and estradiol), no abnormalities on physical examination of the genitourinary system, a total sperm count ≥ 39 million per ejaculate, and ≥ 4% morphologically normal sperm. All included individuals had complete clinical records and follow-up data, and none had known genetic disorders, severe chronic diseases, or a history of fertility-related treatments such as testicular surgery or assisted reproductive technologies. Individuals were excluded if they were younger than 18 or older than 45 years; had chromosomal abnormalities, translocations, Y chromosome microdeletions, or azoospermia; showed scrotal ultrasound abnormalities; had a history of medications known to impair sperm function (e.g., chemotherapeutic agents); or were diagnosed with common infectious diseases such as HIV or syphilis.

### Genetic analysis

Whole-exome sequencing (WES) and bioinformatics analysis were carried out following previously published protocols (12). Briefly, WES was first performed in an infertile family with asthenozoospermia, including the proband (II-2) and his parents (I-1 and I-2). Genomic DNA (200 ng per sample) was captured using the SureSelect Human All Exon V7 kit (Agilent Technologies, 5191−4004) according to the manufacturer’s instructions. Hybridization was carried out overnight at 65 °C for 16 hours to ensure complete probe-target binding. Sequencing libraries were prepared and sequenced on the NovaSeq 6000 platform (Illumina, San Diego, CA) with 150 bp paired-end reads.

Raw sequencing reads were quality-filtered and aligned to the GRCh37 (hg19) reference genome using BWA-MEM (v0.7.17). Variant calling was performed using GATK HaplotypeCaller (v4.2), and duplicate reads were removed using Picard tools (v2.26). Variants were annotated using ANNOVAR (version 2020Jun) and filtered based on the following criteria: minor allele frequency < 0.01 in public databases (gnomAD, 1000 Genomes, ExAC); predicted deleteriousness by SIFT, PolyPhen-2, and CADD scores; and relevance to male infertility according to OMIM, ClinVar, and HGMD databases. Candidate variants were visually validated using the Integrative Genomics Viewer (IGV, v2.16) to confirm read depth and allelic balance.

Selected variants and inheritance patterns were confirmed by Sanger sequencing using the BigDye Terminator v3.1 Cycle Sequencing Kit (Thermo Fisher Scientific, Cat# 4337455) on the Applied Biosystems 3500 DNA Analyzer. Chromatograms were analyzed using Chromas software (v2.6.6).

### Semen analysis

Semen parameters were assessed according to the guideline for the examination and processing of human semen (23). Fresh semen samples were obtained by masturbation after 3–7 days of sexual abstinence and liquefied at 37 °C for 30 min. Progressive motility (%) was analyzed using a computer-assisted semen analysis system (CASA) (IVOS II, Hamilton Thorne). In addition to progressive motility, semen volume, sperm concentration, total sperm count, total motility and immotile sperm rate were evaluated manually according to the WHO 5th edition guidelines.

### Western blot analysis of CAPZA1 in human semen samples

Protein samples were extracted from semen and resolved on a 10% SDS-PAGE gel, then transferred to a PVDF membrane (Millipore, IPVH00010). The membrane was blocked with 5% non-fat milk in Tris-buffered saline with 0.1% Tween-20 (TBST) for 1 hour at room temperature and incubated overnight at 4 °C with a primary antibody against CAPZA1 (Affinity, DF12145, 1:1000). GAPDH (Biosharp, BL006B, 1:1000) was used as a loading control, and band intensities were normalized to GAPDH. After washing, the membrane was incubated with HRP-conjugated goat anti-rabbit IgG secondary antibody (Invitrogen, MA5-56524, 1:1000) for 1 h at room temperature. Protein bands were visualized using the ECL chemiluminescence kit (Thermo Fisher Scientific, 34095), and the intensity of the bands was quantified using ImageJ software (National Institutes of Health).

### Transmission electron microscopy

Sperm samples were initially fixed in 1 mL of 2% glutaraldehyde (Sigma-Aldrich, G5882) and subsequently post-fixed in 1 mL of 1% osmium tetroxide (Electron Microscopy Sciences, 19150). The samples were then dehydrated through a graded ethanol series (50%, 70%, 90%, and 100%) followed by 5 mL of 100% acetone (Thermo Fisher Scientific, A949-4). After dehydration, samples were infiltrated with a mixture of acetone and 2 mL of SPI-Chem embedding resin (SPI Supplies, 90529-77-4), and finally embedded in Epon812 resin (Electron Microscopy Sciences, 14120). Once the resin had polymerized, ultrathin sections (70–90 nm) were prepared using an ultramicrotome (Leica EM UC7). The sections were stained with 1 mL of uranyl acetate (Electron Microscopy Sciences, 22400) and 1 mL of lead citrate (Electron Microscopy Sciences, 17800), and examined using a Talos L120C G2 transmission electron microscope (Thermo Fisher Scientific) at a magnification of ×8,000. Cross-sectional structures of the sperm flagella were observed, with particular attention to the integrity of dynein arms and the fibrous sheath, including defects, loss, or disorganization.

### Isolation of round spermatids from mouse testes

Round spermatids were isolated from mouse testes using enzymatic digestion combined with differential centrifugation (24). C57BL/6 mice were euthanized by an intraperitoneal injection of a lethal dose of sodium pentobarbital (150 mg/kg; Sigma-Aldrich, P010500) (25). This procedure was performed in accordance with ARRIVE guidelines to ensure humane euthanasia (26). Testes were aseptically excised and placed in pre-chilled Dulbecco’s phosphate-buffered saline (Gibco, 14190144). After removing surrounding fat and epididymal tissue, the testes were finely minced with sterile scissors (Fine Science Tools, 14060-09). Tissue fragments were digested with 0.5 mg/mL of collagenase IV (Worthington Biochemical, LS004188) at 37 °C for 10 min in a shaking incubator (Thermo Fisher Scientific, MaxQ 4450), followed by 2 mL of 0.25% trypsin (Thermo Fisher Scientific, 25200056) digestion for an additional 5 min with intermittent pipetting to facilitate cell dissociation. Digestion was terminated by adding 2 mL of fetal bovine serum (FBS, Gibco, 10099141C), and the suspension was filtered through a 200-mesh nylon filter to remove undigested tissue. The filtrate was centrifuged at 300 ×g for 5 min, and the cell pellet was resuspended in DMEM containing 10% FBS. The suspension was incubated at 37 °C in a 5% CO_2_ incubator for 1 h to allow adhesion of Sertoli and other somatic cells. The non-adherent germ cells were collected, centrifuged again at 300 ×g for 5 min, and resuspended in 5 mL of DPBS (Gibco, 14190144). A Percoll density gradient (30%, 50%, and 70%) was used to purify round spermatids via centrifugation at 500 ×g for 20 min. Cells at the 50%-70% interface were collected, washed twice in PBS, and resuspended in an appropriate volume. To verify the identity of round spermatids, immunostaining was performed with polyclonal ACRV1 antibody (Proteintech, 14040-1-AP, 1:400) and polyclonal Prm1 antibody (Proteintech, 15697-1-AP, 1:200). After incubation with the primary antibodies for 1 h at room temperature, cells were washed and incubated with appropriate Alexa Fluor–conjugated secondary antibodies. Nuclei were counterstained with DAPI. Fluorescent images were acquired using a confocal microscope (LSM800, Carl Zeiss).

### Cell transfection

In mouse round spermatids, CAPZA1 knockout (KO-CAPZA1) was achieved using CRISPR-Cas9-mediated gene editing. A single-guide RNA (sgRNA) specifically targeting the CAPZA1 gene (5’-CGGTGCTGGCATAGTCCCTCTGG-3’) was designed and cloned into a plasmid vector encoding the Streptococcus pyogenes Cas9 protein (GeneChem, Shanghai, China). 30 μg/mL of Plasmid DNA (GeneChem) was used for each transfection condition. The plasmid construct was delivered into isolated round spermatids using the Neon Transfection System (Thermo Fisher Scientific, MPK5000) according to the manufacturer’s protocol. A negative control group (NC) was transfected with a plasmid carrying a non-targeting sgRNA sequence (5’-GTGCGAATACGCTCACGGAA-3’). After transfection, cells were incubated at 37 °C in a humidified atmosphere containing 5% CO_2_. Successful genome editing and KO-CAPZA1 efficiency were confirmed by reverse transcription quantitative PCR (RT-qPCR) to assess mRNA levels and by Western blot analysis to evaluate CAPZA1 protein expression. To construct and evaluate the CAPZA1 WT and c.11T>C mutant expression plasmids, the corresponding cDNA sequences were cloned into the pcDNA3.1 vector (GeneChem, Shanghai, China). Mouse round spermatids were transfected with 30 μg/mL of each plasmid (WT or mutant) using the Neon Transfection System. Following 48 h of incubation at 37 °C under 5% CO_2_, cells were lysed and CAPZA1 protein levels were analyzed by Western blot.

### RT-qPCR

Total RNA was extracted from cells or tissues using 1 mL of Trizol reagent (Invitrogen, 15596026) and reverse-transcribed into cDNA using 10 μL of HiScript II Q RT SuperMix (Vazyme, R223-01) per reaction. Quantitative PCR was performed using 10 μL of SYBR Green PCR Master Mix (Bio-Rad, 1725124) with specific primers. Primer sequences were as follows: CAPZA1 forward: 5’-TTCATCACTCATGCACCCCC-3’; reverse: 5’-TCACAGGCGTGAACTGATCC-3’; SLC7A11 forward: 5’-TGGAACGAGGAGGTGGAGAA-3’; reverse: 5’-TGGTGGACACAACAGGCTTT-3’; GAPDH forward: 5’-AGAGTGTTTCCTCGTCCCGT-3’; reverse: 5’-GAGGTCAATGAAGGGGTCGT-3’. To identify key regulators involved in the observed effects, we examined the expression of several genes related to transcriptional regulation, chromatin remodeling, and redox homeostasis in round spermatids. The genes assessed included ATF4, CREBBP, EP300 (encoding the histone acetyltransferase p300), KAT2A, KAT2B, NFE2L2, SLC3A2, and SLC7A11. The primer sequences used for RT-qPCR are provided in **Supplementary Table 1**. qPCR was conducted on the 7500 Real-Time PCR System (Applied Biosystems, 4351106) under the following cycling conditions: initial denaturation at 95 °C for 10 min, followed by 40 cycles of denaturation at 95 °C for 15 s, annealing at 60 °C for 30 s, and extension at 72 °C for 30 s. GAPDH was used as an internal control. Relative mRNA expression levels were calculated using the 2^-ΔΔCt^ method.

### Western blot

Total protein was extracted using lysis buffer (Beyotime, P0013) supplemented with 1% (v/v) protease inhibitor cocktail (Roche, 11873580001). Equal amounts of protein were separated via SDS-PAGE and transferred to PVDF membranes (Millipore, IPVH00010). Membranes were incubated overnight at 4 °C with primary antibodies: CAPZA1 (Affinity, DF12145, 1:1000), p300/CBP (Affinity, AF5487, 1:1000), H3K27ac (ThermoFisher, PA5-85524, 1:1000), SLC7A11 (Bioswamp, RMAB50759, 1:1000), H3 (Servicebio, GB11102, 1:1000), DNAH9 (Biorbyt, orb586345, 1:1000), FSCN1 (Servicebio, GB111025, 1:1000) and GAPDH (Biosharp, BL006B, 1:1000). After washing, membranes were incubated with HRP-conjugated goat anti-rabbit IgG secondary antibody (Invitrogen, MA5-56524, 1:1000) for 1 h at room temperature. Detection was performed using chemiluminescent substrate (Millipore, WBKLS0500) for 5 min. Membranes were exposed to X-ray film and developed. Band intensity was quantified using ImageJ software (version 1.53, National Institutes of Health).

### ChIP-qPCR analysis

Chromatin immunoprecipitation (ChIP) was performed to assess the enrichment of EP300 and H3K27ac in round spermatids from NC and KO-CAPZA1 groups. Briefly, approximately 1 × 10^7^ cells were crosslinked with 1% formaldehyde for 10 min at room temperature, followed by quenching with 125 mM glycine. Cells were then lysed in lysis buffer (1% SDS, 10 mM EDTA, 50 mM Tris-HCl, pH 8.1, 1× protease inhibitors [Sigma-Aldrich, P8340]) and sonicated using a Bioruptor Plus sonicator (Diagenode, UCD-200) for 15 cycles of 30 s on and 30 s off. Chromatin was immunoprecipitated using specific antibodies against EP300 (Abcam, ab16039) or H3K27ac (Cell Signaling Technology, 8173), followed by incubation with Protein A Sepharose beads (Thermo Fisher, 15918) overnight at 4 °C. After washing, the immunoprecipitates were eluted in elution buffer (1% SDS, 0.1 M NaHCO_3_), and crosslinks were reversed by incubating at 65 °C for 4 h. DNA was purified using the QIAquick PCR Purification Kit (Qiagen, 28106) and analyzed by qPCR using the QuantStudio 3 Real-Time PCR System (Thermo Fisher). SYBR Green Master Mix (Applied Biosystems, 4309155) was used for qPCR reactions. Specific primer sequences for the target regions of EP300 and H3K27ac are provided in **Supplementary Table 1**. The enrichment of the target loci was calculated as a percentage of input DNA, and results were normalized to the GAPDH locus as an internal control.

### Intracellular ROS measurement

Intracellular reactive oxygen species (ROS) levels in round spermatids were measured using the fluorescent probe 2’,7’-dichlorofluorescein diacetate (DCFH-DA; Beyotime, S0033S). Briefly, round spermatids were incubated with 10 μM DCFH-DA at 37 °C for 30 min in the dark. After washing three times with PBS to remove excess probe, cells were immediately imaged by confocal microscopy (LSM800, Carl Zeiss) and percentage of DCF-positive cells was quantified using ImageJ.

### ELISA

Cystine levels were measured using a commercial ELISA kit (Abcam, ab285240). Spermatogenic cells were washed twice with PBS and lysed using lysis buffer containing 1% Triton X-100 (Sigma-Aldrich, T8787). The lysates were centrifuged at 10,000 ×*g* for 10 min to remove cellular debris. Protein concentrations in the supernatants were normalized using the Bradford assay (Bio-Rad, 5000006). Standards and samples were added to 96-well plates pre-coated with cystine-specific antibodies, with each sample run in triplicate. A cystine-specific secondary antibody conjugated to an enzyme was added and incubated for 1 h. Wells were washed three times with PBS containing 0.05% Tween-20 to remove unbound antibodies, followed by color development with TMB substrate. The reaction was terminated with 2 M sulfuric acid. Absorbance was measured at 450 nm using a microplate reader (Thermo Fisher Scientific, 51119100), and cystine concentrations were calculated based on the standard curve.

### Thiol quantification assay

Intracellular thiol groups were quantified using 5,5’-dithiobis-(2-nitrobenzoic acid) (DTNB, Ellman’s reagent) (Sigma-Aldrich, D8130). A 10 mM stock solution of DTNB was prepared in anhydrous ethanol and diluted to a final concentration of 0.1 mM in phosphate-buffered saline (PBS, pH 7.4) before use. Round spermatids were lysed, and total protein concentration was determined using a Bradford assay (Bio-Rad) for normalization. To convert disulfide bonds to measurable sulfhydryl (−SH) groups, cell lysates were incubated with 5 mM tris(2-carboxyethyl)phosphine (TCEP) (Thermo Fisher Scientific, 77720) at room temperature for 15 min. Equal volumes of TCEP-treated lysates and DTNB working solution were then mixed and incubated for 15 min at room temperature. The reaction mixtures were transferred to a 96-well plate, and absorbance was measured at 412 nm using a microplate reader. A standard curve was constructed using defined concentrations of reduced glutathione, and DTNB-reactive thiol group concentration was calculated and expressed as nmol/mg protein. Increased absorbance reflects higher thiol content, suggesting altered thiol/disulfide redox status (27).

### Experimental animals

All animal experiments were conducted between February 20, 2025 and June 10, 2025 at the animal facility of Hainan Medical University. All animal procedures were approved by the Institutional Animal Care and Use Committee of Hainan Medical University (Approval No. HYALL-2025-003). This study followed the ARRIVE guidelines to ensure ethical and scientific rigor in study design, animal care, and data reporting. Six specific pathogen-free male C57BL/6 mice, aged 8 weeks, weight 20-25g, were maintained under standard laboratory conditions (temperature: 22-25 °C; humidity: 50%-60%; light/dark cycle: 12 h/12 h), with ad libitum access to food and water. All experimental operations adhered to humane handling principles to minimize animal suffering. Intraperitoneal injection was employed for euthanasia when required. Mice were randomly assigned to KO-CAPZA1 groups and negative control (*n* = 3 per group) using a random number table method. The investigators responsible for data collection and analysis were blinded to group allocation. All animals were euthanized at the end of the experiments for histological analysis.

### Animal model

KO-CAPZA1 mouse model was generated using adeno-associated virus (AAV)-mediated CRISPR-Cas9 gene editing (Obio Technology, Shanghai, China). sgRNA targeting exons 3 of the mouse CAPZA1 gene (5’-CGGTGCTGGCATAGTCCCTCTGG-3’) was designed and cloned into an AAV vector along with the Cas9 gene. A control virus lacking CAPZA1-targeting sgRNA (NC-AAV) (5’-GTGCGAATACGCTCACGGAA-3’) was used as the negative control. Mice were anesthetized using 3% isoflurane (RWD Life Science, R510-22) in oxygen via a precision vaporizer, followed by surgical procedures under full anesthesia (26). After confirming deep anesthesia, surgical sites were sterilized with 70% ethanol and povidone-iodine. A 1.5 cm vertical incision was made in the lower abdomen using sterile scissors to expose the testes, avoiding vascular injury. The testes were gently lifted using fine forceps and immobilized. A total of 10 µL of AAV solution was injected into each testis using a 33-gauge microsyringe (Hamilton, Switzerland), targeting the interstitial space as previous reported (28, 29). The abdominal incision was closed with absorbable sutures, ensuring hemostasis. Following surgery, the mice were kept in a warm environment for 30 min to facilitate virus diffusion and cellular entry. Four weeks after injection, the mice were euthanized by an intraperitoneal injection of an overdose of sodium pentobarbital (150 mg/kg; Sigma-Aldrich, P010500) (25). After ensuring the cessation of respiratory and cardiac activity, dissection was carried out without delay. CAPZA1 protein expression in testicular tissue was assessed by Western blot, and sperm motility was evaluated.

### Mouse sperm analysis

Mouse sperm motility and parameters were assessed using a CASA system (30). The distal cauda epididymis of the right testis was excised and rinsed with warm phosphate-buffered saline, then placed in a microcentrifuge tube containing fresh human tubal fluid medium (Vitrolife, 10128) supplemented with 10% fetal bovine serum (Gibco, 10099141) at 37 °C. The epididymis was then gently punctured with a sterile scalpel to release sperm into the medium. The sperm suspension was diluted to an appropriate concentration using pre-warmed medium. A 10 μL aliquot of the suspension was loaded into the CASA system (IVOS II, Hamilton Thorne, 910-0005-000) for quantitative assessment of sperm parameters.

### Hematoxylin and eosin staining

Mouse testes were photographed, weighed, and fixed in modified Davidson’s fixative (50% distilled water, 30% formaldehyde, 15% ethanol, and 5% glacial acetic acid) for 48 h. After fixation, tissues were dehydrated in a graded ethanol series (70%, 80%, 90%, and 100%) and then cleared in xylene for 1 h. The specimens were embedded in paraffin, sectioned into 4 μm slices, and dewaxed at 65 °C overnight in xylene. Sections were rehydrated through a graded ethanol series (100%, 95%, 70%, and 50%), rinsed in distilled water, and stained with hematoxylin solution (Sigma-Aldrich). After counterstaining and dehydration, slides were cleared with xylene and mounted using DPX mounting medium. Histological observations were performed and recorded using a Leica DMi8 microscope (Leica Microsystems), and representative images were captured.

### Testicular RNA extraction and RT-qPCR

At four weeks after AAV injection, mouse testes were rapidly dissected, snap-frozen in liquid nitrogen, and stored at −80 °C until use. Total RNA was extracted from testicular tissues using TRIzol reagent (Invitrogen, 15596026) according to the manufacturer’s instructions. RNA concentration and purity were assessed using a NanoDrop 2000 spectrophotometer (Thermo Fisher Scientific). Equal amounts of total RNA were reverse-transcribed into cDNA using HiScript II Q RT SuperMix (Vazyme, R223-01). Quantitative real-time PCR was performed using SYBR Green PCR Master Mix (Bio-Rad, 1725124) on a 7500 Real-Time PCR System (Applied Biosystems, 4351106). The expression levels of DNAH9 and FSCN1 were quantified, with GAPDH used as an internal control. Relative mRNA expression levels were calculated using the 2^-ΔΔCt^ method. Primer sequences were as follows: DNAH9 forward: 5’- CTTGAGTGCCTCCTGACAAAGG -3’; DNAH9 reverse: 5’- GCCACCATTTGCTGAACTCTGC -3’; FSCN1 forward: 5’- AGGACCAACGCTACAGTGTG -3’; FSCN1 reverse: 5’- AACAGGAGGGACTGACGAGA -3’; GAPDH forward: 5’- GTGTTCCTACCCCCAATGTGT -3’; GAPDH reverse: 5’- ATTGTCATACCAGGAAATGAGCTT -3’.

### Immunofluorescence staining

Sperm samples were air-dried on glass slides and fixed with 200 μL of 4% paraformaldehyde (Solarbio, P0099). After fixation, slides were washed three times with PBST (0.1% Tween-20 in PBS) for 5 min each and blocked with 1% BSA/PBST for 1 h. Samples of round spermatids and mature sperm were incubated overnight at 4 °C with primary antibodies: DNAH9 (Invitrogen, PA5-45744, 1:50), FSCN1 (Invitrogen, 6624-MSM3-P1ABX, 1:100) and CAPZA1 (Affinity, DF12145, 1:200). On the next day, after washing, round spermatids were incubated with Alexa Fluor 594-conjugated anti-rabbit IgG (Invitrogen, A-11012, 1:1000) to detect DNAH9 (red), and Alexa Fluor 488-conjugated anti-mouse IgG (Invitrogen, A-11001, 1:1000) to detect FSCN1 (green). For mature sperm, the labeling was reversed: Alexa Fluor 488 for DNAH9 (green), and Alexa Fluor 594 for FSCN1 (red). CAPZA1 was detected using Alexa Fluor 488–conjugated goat anti-rabbit IgG (H+L) (Invitrogen, A-11008, 1:1000). All secondary antibodies were incubated for 2 h at room temperature. Slides were rinsed with PBS and mounted using 50 μL of glycerol mounting medium (Beyotime, P0126). Imaging was performed using an LSM800 confocal microscope (Carl Zeiss AG) with ZEN2013 software. Nuclei were counterstained with DAPI.

### Scanning electron microscopy observations of sperm morphology

The spermatozoa samples were fixed in 2.5% glutaraldehyde (Servicebio, G1102) at 4 °C overnight, washed with PBS, and post-fixed with 1% osmium tetroxide (Sigma-Aldrich, 75632) for 1 hour at room temperature. After graded ethanol dehydration and critical-point drying (Leica EM CPD300), sperm were mounted onto metal stubs, sputter-coated with gold using an ion sputter coater (Hitachi MC1000), and observed under a scanning electron microscope (Hitachi SU8100). Images were captured at both low and high magnifications to evaluate overall sperm morphology and detailed tail ultrastructure.

### TEM of mouse sperm

To evaluate the ultrastructural integrity of sperm flagella in KO-CAPZA1 and control mice, transmission electron microscopy (TEM) was performed. Sperm samples were collected from the cauda epididymis and initially fixed in 2% glutaraldehyde (Sigma-Aldrich, G5882) at 4 °C for 2 h. Samples were then post-fixed in 1% osmium tetroxide (Electron Microscopy Sciences, 19150) for 1 h at room temperature. Following fixation, sperm were dehydrated in graded ethanol solutions (50%, 70%, 90%, and 100%), transitioned through propylene oxide, and embedded in Epon812 resin (Electron Microscopy Sciences, 14120). Ultrathin sections (70–90 nm) were obtained using an ultramicrotome (Leica EM UC7), stained with uranyl acetate and lead citrate, and examined using a Talos L120C G2 transmission electron microscope (Thermo Fisher Scientific) at a magnification of ×10,000. Cross-sectional and longitudinal views of sperm flagella were analyzed for axonemal “9 + 2” structure, outer dense fibers, fibrous sheath, and mitochondrial sheath abnormalities.

### Statistical analysis

All data were analyzed using Prism 10.0 statistical software (GraphPad Software, San Diego, CA, USA). Quantitative data are presented as mean ± standard deviation (SD). Comparisons between two groups were performed using independent-samples t-tests or Mann-Whitney U tests, as appropriate. One-way analysis of variance (ANOVA) followed by Tukey’s HSD *post hoc* test was used for multiple group comparisons. The correlation between CAPZA1 expression and progressive motility was analyzed using Spearman’s rank correlation test. *p* < 0.05 was considered statistically significant.

## Results

### CAPZA1 mutation leads to protein deficiency and impairs sperm progressive motility and flagellar ultrastructure

**Figure 1:**
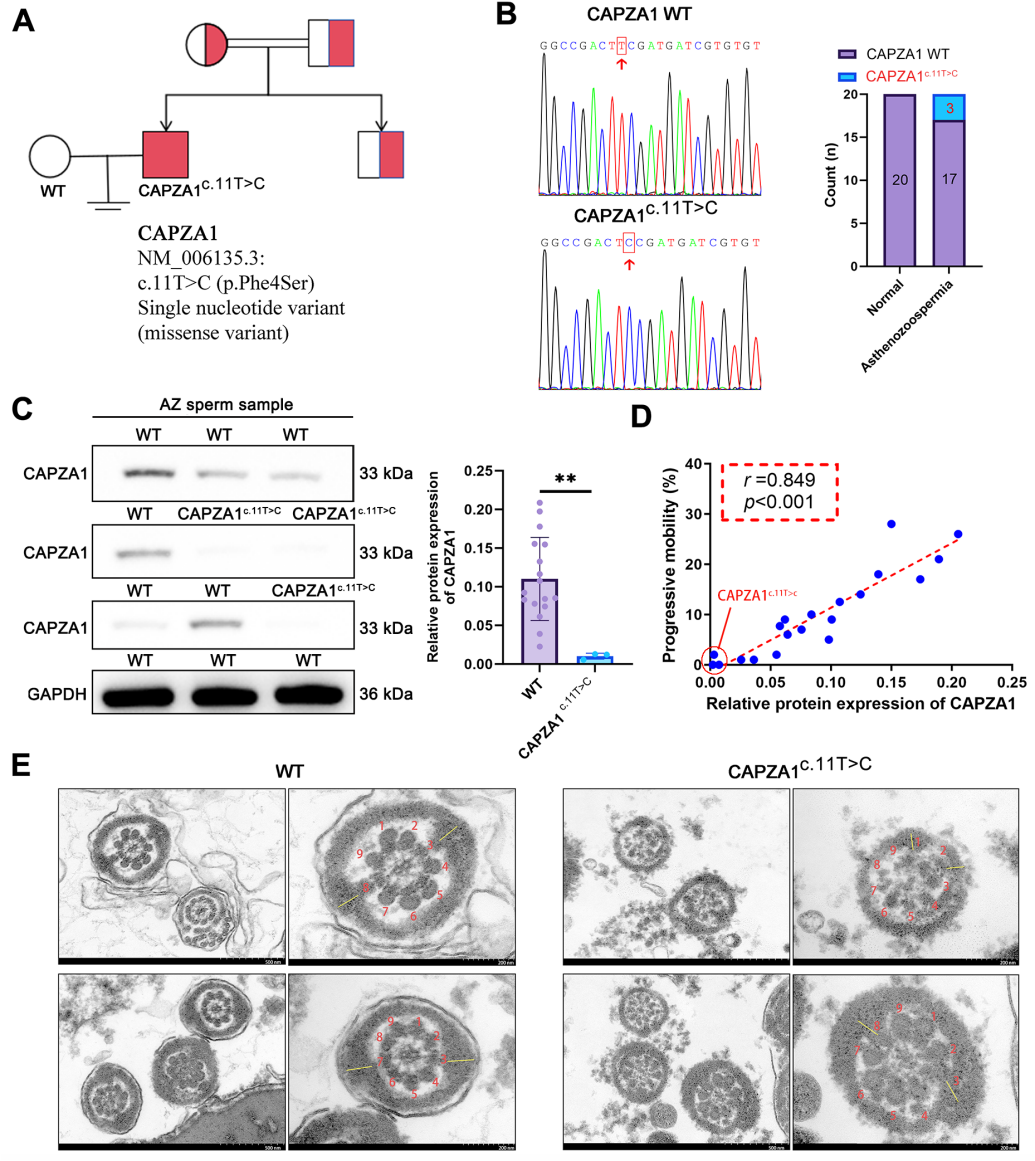
CAPZA1 mutation leads to protein deficiency and impairs sperm progressive motility and flagellar ultrastructure. **(A)** Pedigree analysis was performed using whole-exome sequencing in an infertile family. Red-filled squares indicate infertile males, with the nucleotide variant information shown below the pedigree. **(B)** Sanger sequencing chromatograms show wild-type (WT) and mutant (c.11T>C) sequences, with the mutation site highlighted by red boxes and indicated by red arrows. The bar graph represents the number of individuals carrying the WT or CAPZA1^c.11T>C^ in normal (*n* = 20) and asthenozoospermic (*n* = 20) groups. **(C)** Western blot analysis of CAPZA1 expression in non-mutant asthenozoospermic patients (WT) (*n* = 17) and mutant asthenozoospermic patients (CAPZA1^c.11T>C^) (*n* = 3). **(D)** Spearman correlation between CAPZA1 protein levels and progressive motility. **(E)** Transmission electron microscopy images of sperm flagella in WT and CAPZA1^c.11T>C^ sperm. The red numbers denote nine peripheral microtubule doublets, the yellow dash represents longitudinal columns. Scale bars = 500 nm, enlarged = 200 nm. Data are presented as mean ± SD (*n* = 3). ^**^p < 0.01 compared with WT group.

WES was performed in an infertile family, and a rare homozygous missense variant in CAPZA1 (NM_006135.3: c.11T>C, p.Phe4Ser) was identified and subsequently confirmed by Sanger sequencing. Representative sequencing chromatograms are shown in **Figures 1B**, with the mutation site indicated by red arrows. Pedigree analysis revealed that this variant follows an autosomal recessive inheritance pattern, with the proband being homozygous and both parents being asymptomatic heterozygous carriers (**Figures 1A, B**). This variant was detected in 3 of 20 individuals with asthenozoospermia, but absent in all 20 controls (**Figure 1B**). Western blot analysis of sperm samples demonstrated a significant reduction in CAPZA1 protein expression in asthenozoospermic patients with the CAPZA1c.11T>C mutation compared to wild-type asthenozoospermic patients (**Figure 1C**). Immunofluorescence analysis further corroborated this conclusion and revealed that CAPZA1 is mainly localized to the sperm head, whereas only weak fluorescence was observed along the flagellum (**Supplementary Figure 1**). A strong positive correlation was observed between CAPZA1 protein expression and progressive sperm motility (r = 0.849, p < 0.001; **Figure 1D**). TEM revealed that wild-type sperm flagella exhibited a typical 9 + 2 axonemal ultrastructure with symmetrical organization. In contrast, sperm from the CAPZA1c.11T>C mutant displayed disorganized and asymmetric axonemal structures, with irregular microtubule arrangement and disruption of longitudinal columns (**Figure 1E**).

Consistent with the diagnosis of asthenozoospermia, statistical analysis revealed significantly lower total motility and progressive motility in the asthenozoospermic group compared to controls (both p < 0.001), along with a significantly higher proportion of immotile sperm (p < 0.001), as shown in **Table 1**. In contrast, no significant differences were observed in other semen parameters, including ejaculate volume, sperm concentration, total sperm count, non-progressive motility, or morphologically normal sperm percentage. Notably, a modest but statistically significant difference was detected in semen pH between the two groups (p = 0.030), while age, BMI, and days of abstinence were comparable. These findings indicate that the CAPZA1 mutation results in decreased protein expression and disrupts both progressive motility and the structural integrity of flagellar components.

**Table 1:** Clinical data of normal and asthenozoospermic semen samples.

Characteristics	Normal (*n* = 20)	Asthenozoospermic (*n* = 20)	*p*
Age (years) Body mass index (BMI)	33.5 (31.75, 35) 22.06 ± 1.67	33 (31, 37.25) 22.1 ± 1.79	0.871 0.935
Days of abstinence	3 (3, 6.25)	4.5 (3, 5)	0.807
Ejaculate volume (ml)	3.365 ± 1.2504	3.785 ± 1.4299	0.329
Sperm concentration (10^6^/ml)	115.03 ± 57.533	113.51 ± 93.427	0.951
Total sperm count (10^6^)	353.77 ± 163.43	346.56 ± 136.64	0.880
Total motility (%)	53.6 ± 8.9877	17.335 ± 11.286	< 0.001
Non-progressive motility (NP) (%)	7.65 ± 2.3458	7.525 ± 7.1955	0.942
Progressive motility (PR) (%)	45.95 ± 8.4572	9.81 ± 8.5311	< 0.001
Immotile sperm rate (%)	46.4 ± 8.9877	82.665 ± 11.286	< 0.001
pH	7.2 (7.2, 7.425)	7.2 (7.2, 7.2)	0.030
Morphologically normal sperm (%)	9.415 ± 4.2112	7.44 ± 2.6361	0.085

### CAPZA1 knockout suppresses the p300/SLC7A11 pathway and disrupts cystine-dependent thiol–disulfide redox balance in mouse round spermatids

To investigate the early-stage molecular mechanisms of CAPZA1 in spermatogenesis, round spermatids were isolated from mouse testes. Immunofluorescence staining was performed to confirm the identity of the cells using ACRV1 (green) and Prm1 (red) as markers. The results showed robust cytoplasmic ACRV1 staining, weak Prm1 immunofluorescence, and round DAPI-stained nuclei characteristic of round spermatids. Merged images confirmed co-expression of both markers, validating the identity of the isolated cell population (**Supplementary Figure 2**). Gene expression analysis revealed that the levels of CREBBP (*p* < 0.01), EP300 (*p* < 0.001), KAT2A (*p* < 0.01), KAT2B (*p* < 0.05), and SLC7A11 (*p* < 0.001) were significantly reduced in KO-CAPZA1 spermatids compared to the NC group. In contrast, the expression of ATF4, SLC3A2, and NFE2L2 showed no statistically significant differences between the two groups (**Supplementary Figure 3**). These results suggest that KO-CAPZA1 affects key gene expression in round spermatids.

**Figure 2:**
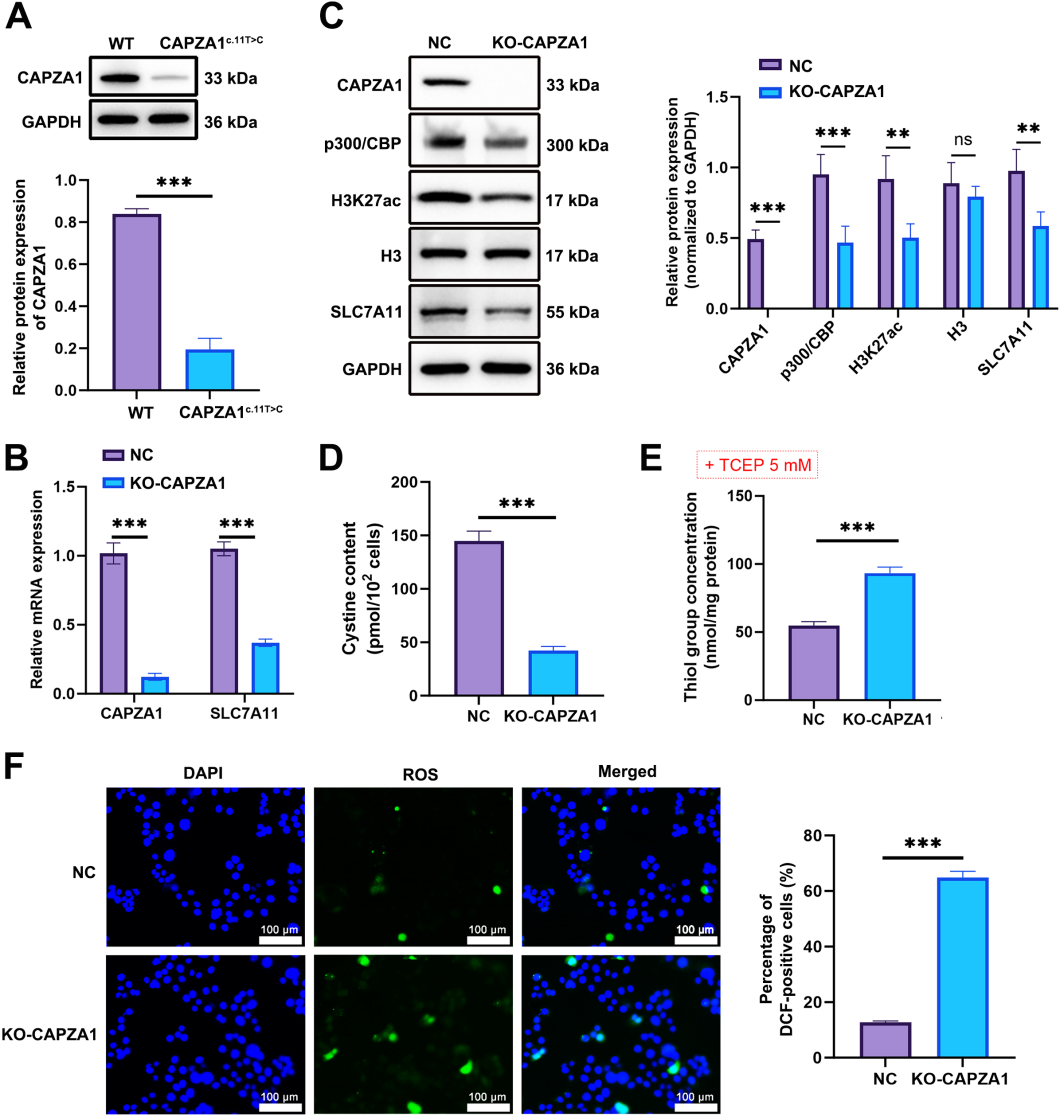
CAPZA1 knockout suppresses the p300/SLC7A11 pathway and disrupts cystine-dependent thiol–disulfide redox balance in mouse round spermatids. **(A)** Construct CAPZA1 wild-type **(WT)** and CAPZA1^c.11T>C^ mutant vectors, and detect CAPZA1 protein expression after transfection. **(B)** RT-qPCR analysis showing the relative mRNA expression level of CAPZA1. **(C)** Western blot analysis of CAPZA1, p300/CBP, H3K27ac, SLC7A11 and H3 relevant protein expression. **(D)** ELISA quantification of intracellular cystine levels. **(E)** Thiol quantification assay. **(F)** Intracellular reactive oxygen species (ROS) levels measured using the DCFH-DA fluorescent probe. Scale bar = 100 μm. Data are presented as mean ± SD (*n* = 3). **p < 0.01, ^***^p < 0.001 compared with NC group. TCEP: tris (2-carboxyethyl) phosphine.

Transfection with the CAPZA1c.11T>C mutant construct resulted in significantly reduced CAPZA1 protein expression compared to cells transfected with the wild-type construct, confirming the functional impact of this mutation (**Figure 2A**). Furthermore, after isolating round spermatids, CAPZA1 was knocked out using sgRNA-mediated Cas9 editing (KO-CAPZA1), with a non-targeting control group (NC) included. mRNA levels of CAPZA1 and SLC7A11 were measured by RT-qPCR, and protein levels of pathway components (p300, SLC7A11, H3K27ac) were analyzed by Western blot. Cystine content was quantified by ELISA, and thiol–disulfide redox balance was evaluated using DTNB after TCEP treatment to detect thiol groups. Results showed significantly reduced mRNA and protein expression of CAPZA1 and SLC7A11 in the KO-CAPZA1 group compared to NC group (p < 0.001; **Figures 2B, C**). Additionally, protein levels of p300/CBP and H3K27ac were markedly decreased (p < 0.001; **Figure 2C**). To further confirm the epigenetic regulation of CAPZA1 on chromatin activity, ChIP-qPCR analysis showed a marked decrease in the enrichment of EP300 and H3K27ac at the target loci in KO-CAPZA1 round spermatids (p < 0.001) (**Supplementary Figure 4**). ELISA demonstrated a significant reduction in cystine content in KO-CAPZA1 cells (p < 0.001; **Figure 2D**). Following TCEP treatment, DTNB-reactive thiol group concentration was significantly elevated in KO-CAPZA1 cells (p < 0.001; **Figure 2E**), consistent with disturbed redox homeostasis. Intracellular ROS levels were significantly elevated in KO-CAPZA1 round spermatids compared with NC cells (**Figure 2F**). These findings suggest that CAPZA1 deficiency impairs the p300/SLC7A11 axis, leading to reduced cystine uptake and dysregulated thiol-based redox buffering capacity, thereby elevating intracellular ROS levels and affecting redox homeostasis in spermatogenic cells.

### CAPZA1 knockout reduces expression and alters localization of dynein arm protein DNAH9 and fibrous sheath protein FSCN1 in mouse round spermatids

**Figure 3:**
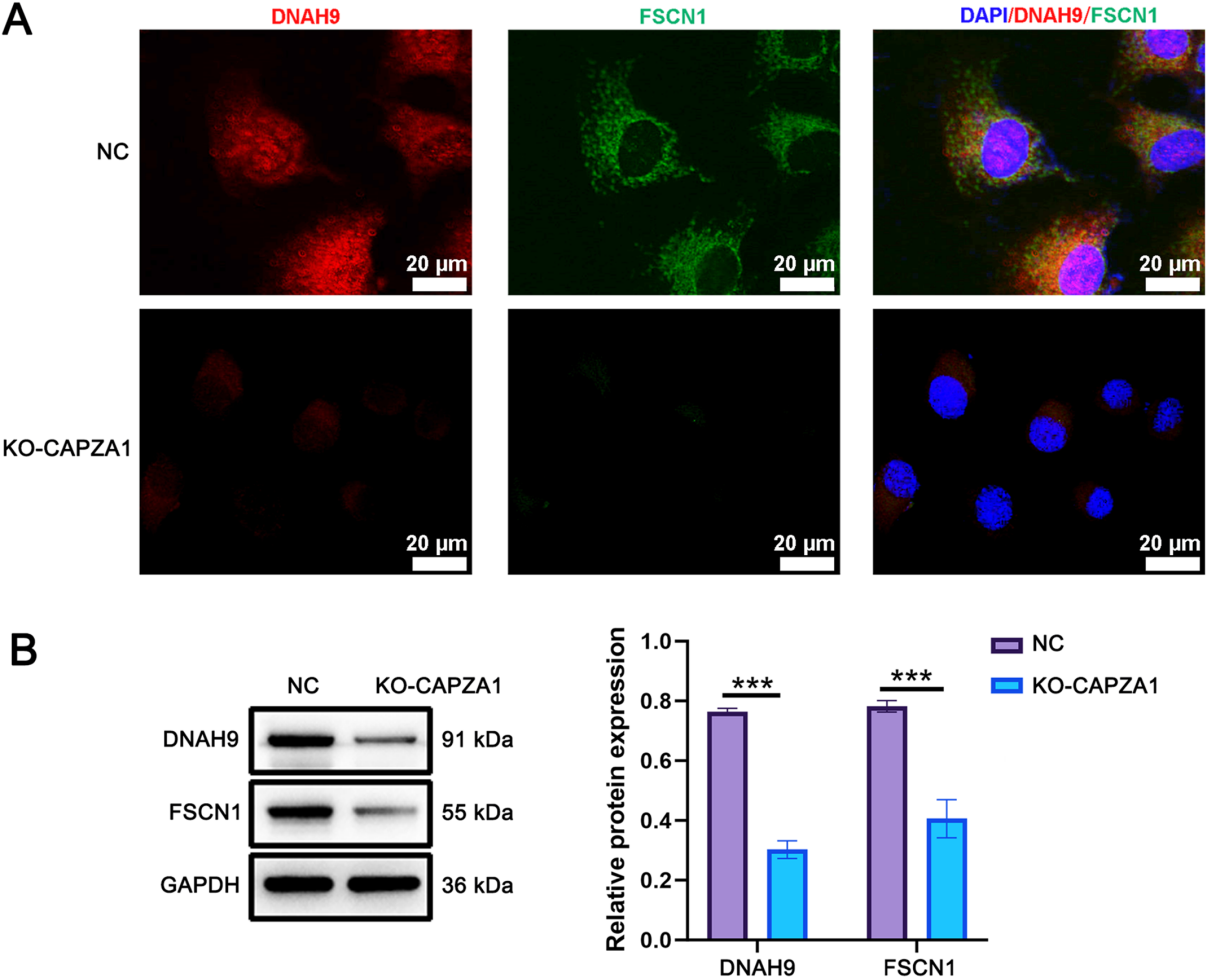
CAPZA1 knockout reduces expression and alters localization of DNAH9 and FSCN1 in mouse round spermatids. **(A)** Immunofluorescence staining shows expression and distribution of dynein arm protein DNAH9 and fibrous sheath protein FSCN1 in round spermatids. Scale bar = 20 μm. **(B)** Western blot analysis of DNAH9 and FSCN1 relevant protein expression. Data are presented as mean ± SD (*n* = 3). ^***^p < 0.001 compared with NC group.

Immunofluorescence staining was used to detect the expression and localization of DNAH9 (dynein arm protein, red) and FSCN1 (fibrous sheath protein, green) in round spermatids. In the NC group, both proteins exhibited robust and evenly distributed cytoplasmic signals. In contrast, KO-CAPZA1 cells showed reduced fluorescence intensity for both DNAH9 and FSCN1 (**Figure 3A**). DAPI staining indicated intact nuclei in both groups. The negative control immunofluorescence images (using only secondary antibody) for round spermatids are shown in **Supplementary Figure 5**. Additionally, protein levels of DNAH9 and FSCN1 were markedly decreased in KO-CAPZA1 group (p < 0.001; **Figure 3B**). These results suggest that CAPZA1 deletion leads to reduced expression and altered subcellular distribution of key structural proteins, indicating a potential role of CAPZA1 in the initiation of sperm flagellum biogenesis.

### CAPZA1 deletion in mouse testis impairs sperm motility

**Figure 4:**
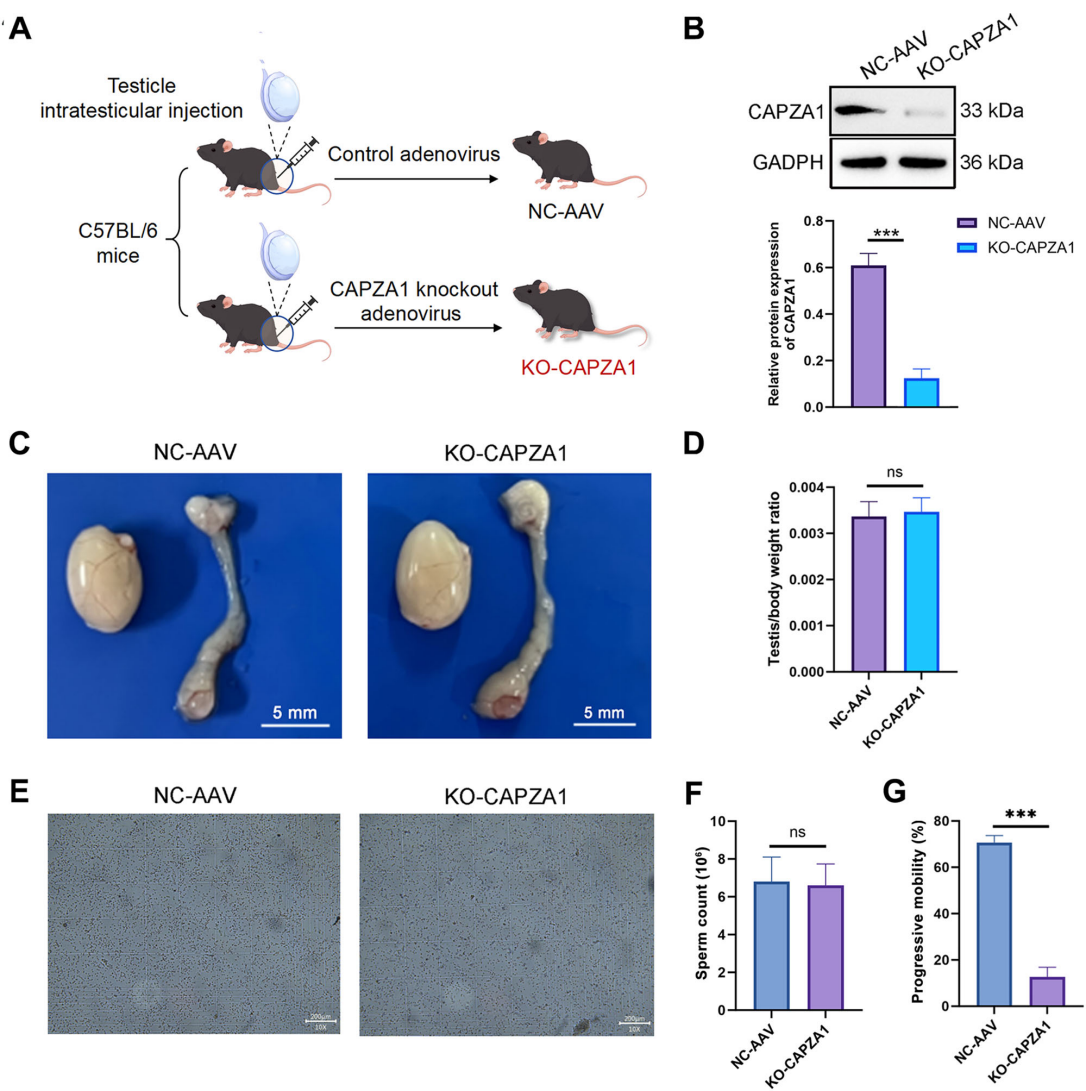
CAPZA1 deletion in mouse testis impairs sperm motility. **(A)** Schematic of experimental grouping. **(B)** Western blot validation of CAPZA1 protein expression in testicular tissue. **(C)** Representative images of mouse testes and epididymis. Scale bar = 5 mm. **(D)** Testis-to-body weight ratio statistics. **(E)** Microscopic imaging of sperm count. Scale bar = 200 μm. **(F)** Quantitative analysis of total sperm count. **(G)** Analysis of sperm progressive motility. Data are presented as mean ± SD (*n* = 3). ^***^p < 0.001 compared with NC-AAV group, “ns” indicates no significant difference between groups.

**Figure 4A** outlines the experimental design. CAPZA1 expression was significantly reduced in the testicular tissue of KO-CAPZA1 mice (**Figure 4B**). No significant differences in testicular morphology or testis-to-body weight ratio were observed between the KO-CAPZA1 and NC-AAV groups (**Figures 4C, D**), suggesting that testicular development was not affected by CAPZA1 deletion. Additionally, although total sperm count showed a slight decrease in the KO-CAPZA1 group, the difference was not statistically significant compared to the NC-AAV group (**Figures 4E, F**). In contrast, the progressive motility of sperm was significantly decreased in the KO-CAPZA1 group (*p* < 0.001; **Figure 4G**), indicating impaired sperm motility and a phenotype resembling asthenozoospermia.

### CAPZA1 knockout disrupts sperm flagellar structure and reduces expression of dynein arm and fibrous sheath proteins

**Figure 5:**
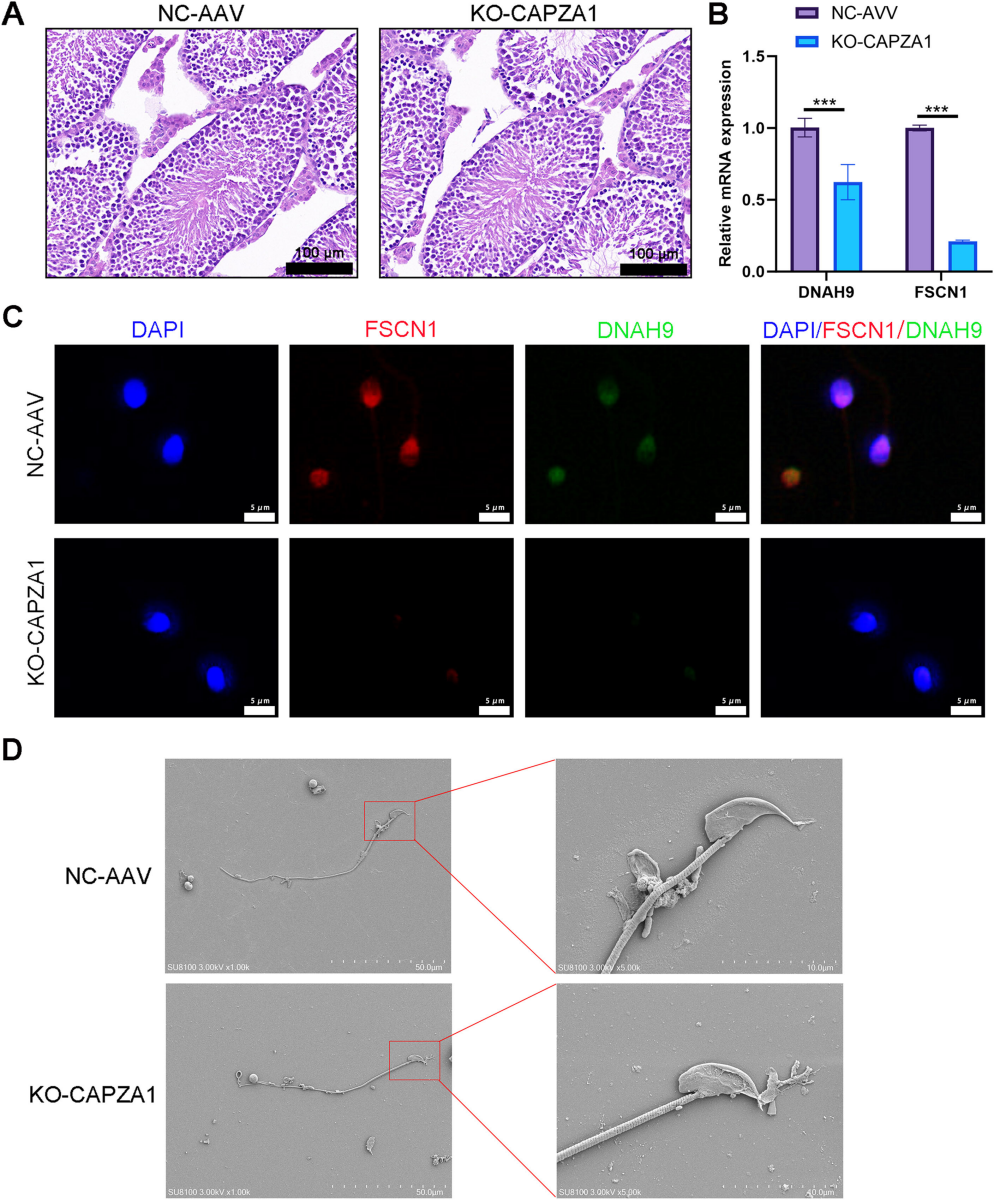
CAPZA1 knockout reduces expression of dynein arm and fibrous sheath proteins in mouse sperm. **(A)** Hematoxylin and eosin (H&E) staining of testicular tissue in KO-CAPZA1 mice (*n* = 3). Scale bar = 100 μm. **(B)** RT-qPCR analysis of DNAH9 and FSCN1 mRNA expression in testicular tissues (*n* = 3). **(C)** Immunofluorescence analysis of sperm smears shows localization of DNAH9 (dynein arm protein, green) and FSCN1 (fibrous sheath protein, red) in sperm (*n* = 3). Scale bar = 5 μm. **(D)** Scanning electron microscopy images demonstrate the overall sperm morphology and head structure appear. Scale bar (left panel) = 50 μm; enlarged region (right panel) = 10 μm. ***p < 0.001.

**Figure 6:**
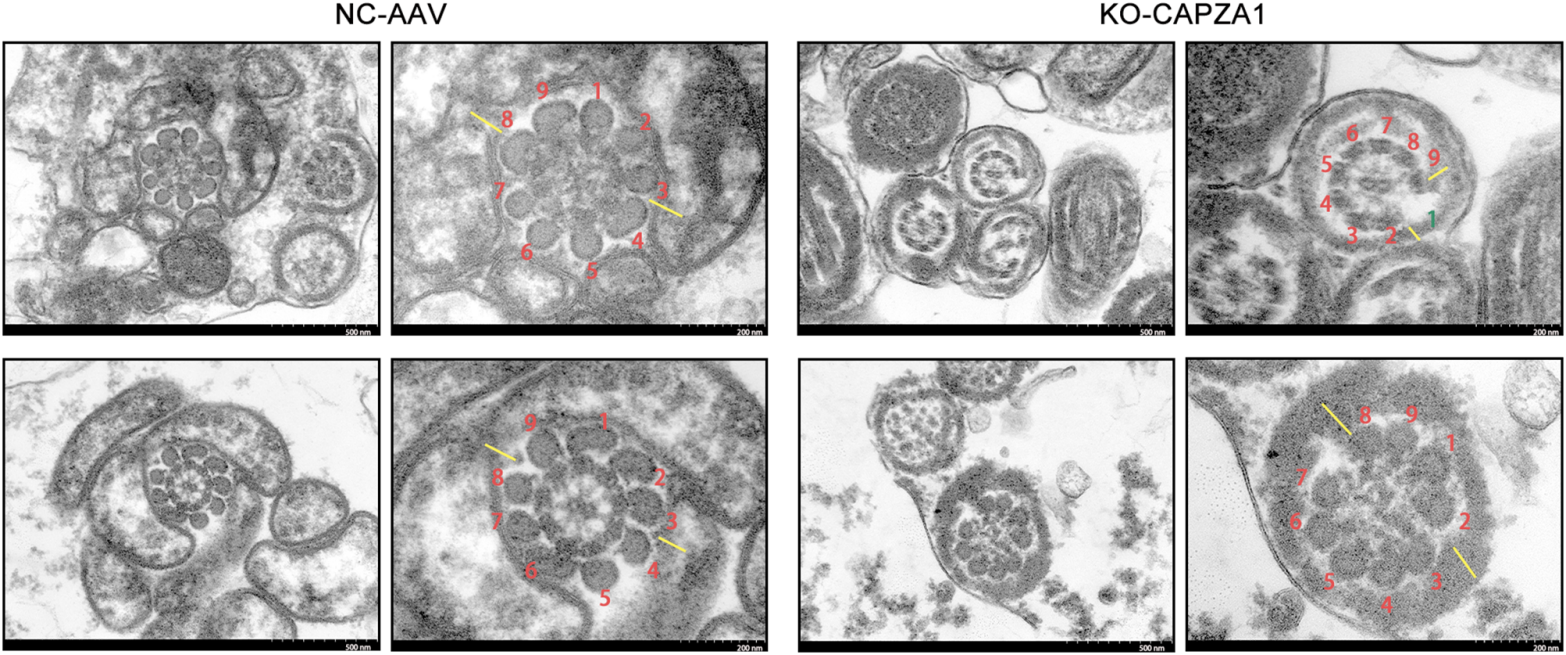
Transmission electron microscopy analysis of sperm flagellar ultrastructure in CAPZA1 knockout mice. Scale bar = 500 nm, enlarged = 200 nm.

H&E staining revealed no significant histological abnormalities in the testicular architecture of KO-CAPZA1 mice when compared to the NC-AAV group, with seminiferous tubules retaining a normal structural arrangement (**Figure 5A**). RT-qPCR results showed that the mRNA expression levels of DNAH9 and FSCN1 were significantly decreased in the testes of KO-CAPZA1 mice compared with the NC-AAV group (**Figure 5B**). However, immunofluorescence staining of sperm smears showed reduction in the expression of FSCN1 (red) and DNAH9 (green) in the KO-CAPZA1 group (**Figure 5C**). DAPI staining confirmed that nuclear morphology remained normal in both groups. The negative control immunofluorescence images (using only secondary antibody) for mouse sperm samples are shown in **Supplementary Figure 5**. Furthermore, SEM showed that while the overall sperm morphology and head structure appeared comparable between the two groups, KO-CAPZA1 sperm exhibited notable structural abnormalities in the tail region (**Figure 5D**). In the NC-AAV group, most sperm flagella displayed intact “9 + 2” axonemal structures. In contrast, sperm from KO-CAPZA1 mice exhibited various abnormalities, including disorganized axonemes, loss of central microtubule pairs, and disrupted outer dense fibers (**Figure 6**), indicating that KO-CAPZA1 leads to flagellar disassembly and impaired axonemal integrity.

## Discussion

WES has emerged as a powerful diagnostic tool for elucidating the genetic basis of male infertility, particularly in cases of asthenozoospermia, by enabling the identification of pathogenic variants involved in sperm flagellar assembly and motility regulation (31–33). In this study, we identified a homozygous CAPZA1^c.11T>C^ (p.Phe4Ser) variant that follows an autosomal recessive inheritance pattern, accompanied by markedly reduced protein expression in spermatozoa and a strong positive correlation between CAPZA1 expression and progressive sperm motility. Several studies have identified pathogenic mutations in genes encoding axonemal outer dynein arm (ODA) proteins through WES, including DNAH10, DNAH11, DNAH9, and DNAH8. These mutations have been consistently associated with severe morphological abnormalities of sperm flagella (MMAF) and impaired motility, often resulting in male infertility (34–37). These mutations typically lead to reduced or absent protein expression, along with ultrastructural defects of the flagella. Among the most prominent abnormalities is the loss of outer dynein arms (ODAs). In contrast, the phenotype observed in CAPZA1-mutant sperm presents distinct features. These include asymmetrical fibrous sheath structures, disorganized filament alignment, partial dynein arm loss. This last feature is rarely observed in previously described DNAH mutations. Such findings suggest that CAPZA1 may serve broader functions beyond axonemal structure maintenance, potentially contributing to nuclear remodeling during spermiogenesis. *In vivo* deletion of CAPZA1 in mouse testes further supported this conclusion, as it led to impaired sperm progressive motility without altering testicular morphology or sperm count, and was associated with disorganized axonemal ultrastructure and reduced expression of dynein arm and fibrous sheath proteins.

The sperm flagellum, with its canonical “9 + 2” axonemal configuration, relies on coordinated dynein-driven bending to generate progressive motility (38). Disruption of critical structural components such as dynein arms, radial spokes, or associated regulatory complexes commonly impairs motility and leads to morphological deformities of the flagellum (39). Notably, the structural abnormalities observed in CAPZA1-mutant sperm partially resemble those reported in individuals with pathogenic variants in axonemal genes such as DNALI1 or DNAH17, although distinct features such as disrupted chromatin condensation suggest a broader functional role for CAPZA1 beyond classical dynein-related mechanisms (39, 40). Recent cryo-electron tomography studies have uncovered molecular asymmetries within the axoneme that are essential for generating non-planar, asymmetric beating patterns, which facilitate sperm navigation through the female reproductive tract (41). The observed asymmetric fibrous sheath and axonemal disorganization in CAPZA1-deficient sperm may disrupt these specialized waveforms, thereby underscoring the structural and functional role of CAPZA1 in maintaining flagellar integrity (42).

Moreover, the cytoskeleton plays a critical role in spermiogenesis by orchestrating processes such as acrosome formation, nuclear condensation, and flagellar assembly (43, 44). CAPZA1, as an actin-capping protein, regulates actin polymerization dynamics, which are essential for cytoplasmic clearance and proper flagellum development (15). DNAH9 is involved in sperm motility, and defects in DNAH9 lead to loss of dynein arms, impairing flagellar movement in asthenozoospermia (45). FSCN1 regulates actin filament bundling, crucial for sperm tail structure and motility (46). In this study, we observed altered expression and mislocalization of cytoskeletal proteins such as DNAH9 and FSCN1 in CAPZA1-deficient sperm, suggesting that the loss of CAPZA1 may lead to cytoskeletal disruption and impaired spermatid maturation. Supporting this, previous transcriptomic analyses in patients with non-obstructive azoospermia have reported downregulation of CAPZA1 along with other cytoskeletal and adhesion-related genes (15). Taken together, our findings expand the genetic landscape of asthenozoospermia by identifying CAPZA1, a non-dynein actin-regulatory protein, as a novel contributor to both flagellar dysfunction and chromatin abnormalities in sperm. These results underscore the complex and heterogeneous pathogenic mechanisms that underlie male infertility.

CAPZA1 expression was found to be mainly localized to the sperm head, suggesting its potential involvement in gene regulation. At the epigenetic level, CAPZA1 appears to play a critical role for maintaining the active chromatin landscape required for spermatid differentiation. Specifically, KO-CAPZA1 resulted in the downregulation of the histone acetyltransferase p300, leading to a global reduction in H3K27ac levels and decreased occupancy of p300/H3K27ac at specific gene promoters. p300/CBP-catalyzed H3K27ac is widely used as a hallmark of active enhancers and is also enriched at active promoters, and p300/CBP activity has been linked to the establishment of an open, accessible chromatin state at regulatory elements (47). Its suppression in CAPZA1-deficient spermatids suggests a disruption in the gene expression programs governing cytoskeletal assembly. While round spermatids undergo global transcriptional silencing, they retain specific active chromatin domains enriched in both H3K27ac and H3K4me3, which collaboratively regulate genes involved in flagellar biogenesis and acrosome development (48, 49). The loss of H3K27ac in our model implies that this permissive chromatin state is compromised.

A key downstream consequence of this epigenetic dysregulation is the impairment of redox homeostasis. We observed that the downregulation of the CAPZA1–p300 axis directly correlated with reduced expression of the cystine transporter SLC7A11. SLC7A11 is pivotal for intracellular cystine uptake, which is the limiting step for cysteine synthesis and subsequent disulfide (–S–S–) bond formation (50, 51). Dynamic thiol–disulfide exchanges are essential for protein folding and structural stability during sperm maturation. In line with this, CAPZA1-deficient spermatids exhibited elevated free thiol content and altered thiol–disulfide redox balance. Previous studies support this mechanism, showing that SLC7A11 deficiency compromises the mechanical stiffness of sperm flagella and leads to asthenospermia or ferroptosis (52). Thus, our findings propose a model where CAPZA1 modulates p300-dependent H3K27ac to sustain Slc7a11 expression, thereby ensuring the redox balance required for the structural integrity of developing sperm.

Consistent with this, CAPZA1-deficient round spermatids showed reduced expression and altered localization of DNAH9 and FSCN1, two key cytoskeletal proteins involved in dynein arm structure and fibrous sheath assembly, respectively. This suggests that CAPZA1 not only regulates cytoplasmic actin filament dynamics but may also influence nuclear gene expression via epigenetic mechanisms. Notably, proteomic analyses have demonstrated that proteins downregulated in asthenozoospermia are involved in nucleosome assembly, protein folding, and flagellar function (53), all of which were affected in our model. Testicular metabolomic and transcriptomic studies have revealed that round spermatids undergo significant metabolic adaptations influencing chromatin modifiers and histone acetylation enzymes (54). CAPZA1’s role in coordinating redox balance, cytoskeletal protein expression, and chromatin regulation may therefore reflect an integrative mechanism required for proper spermatid differentiation. While causality between these processes remains to be fully delineated, our findings support a model in which CAPZA1 serves as a central regulator of cytoskeletal integrity, redox metabolism, and transcriptional programming during spermiogenesis.

We initially isolated round spermatids from mouse testes and performed CRISPR-Cas9–mediated KO-CAPZA1 to investigate its role during early spermatogenesis. Functional assays revealed altered thiol–disulfide redox balance, and downregulation of key structural and epigenetic regulators, highlighting CAPZA1’s role in round spermatid differentiation. Complementary in vivo experiments using CAPZA1-knockout mouse testes further demonstrated impaired sperm motility and protein expression. While this dual approach allowed stage-specific investigation, it should be noted that round spermatids and mature sperm differ in regulatory mechanisms, and the findings in early germ cells may not fully reflect CAPZA1’s role in mature sperm function (55). To achieve in vivo gene editing, we employed direct intratesticular AAV-mediated delivery of CRISPR-Cas9, a rapid and localized alternative to traditional germline knockout models. Unlike embryonic CRISPR/Cas9 editing, which requires time-consuming breeding and resource investment (11, 56–58), this method enables efficient gene disruption in testicular cells without altering tissue architecture, as confirmed by histological analysis. While not heritable, this approach is suitable for evaluating gene function in a cell-type– and stage-specific context.

This study has several limitations. First, the lack of rescue experiments limits causal interpretation of the observed phenotypes. Second, findings based on murine models may not fully translate to humans due to species differences. Moreover, our study did not include CAPZA1 protein measurements in a healthy control group; therefore, we are currently unable to determine whether CAPZA1 expression in asthenozoospermic individuals without the variant is lower than that in normal controls. Additionally, the clinical sample size was limited, and further validation in larger cohorts of asthenozoospermia patients is needed. Moreover, the absence of temporal analysis across spermatogenic stages restricts understanding of CAPZA1’s dynamic role. Future studies should employ germline knockout models and longitudinal analysis to define CAPZA1’s functions during sperm maturation. Investigating whether metabolic or epigenetic modulation can rescue CAPZA1 deficiency may also offer new therapeutic avenues for male infertility. Although our data suggest that CAPZA1 deficiency is associated with reduced p300 activity and SLC7A11 expression, rescue experiments will be required to definitively establish a causal CAPZA1–p300/SLC7A11 axis.

## Conclusion

This study identifies a homozygous missense mutation in CAPZA1 associated with asthenozoospermia. Functional analyses demonstrate that CAPZA1 deficiency is associated with reduced sperm motility, disrupted flagellar ultrastructure, and downregulation of the p300/SLC7A11 pathway components in round spermatids. These findings suggest that CAPZA1 is essential for maintaining sperm structural integrity and redox balance during spermatogenesis. This work provides new insight into the genetic basis of male infertility.

## Data Availability

WES data have been uploaded to the SRA database. Link: https://www.ncbi.nlm.nih.gov/bioproject/PRJNA1426168. Further data are available from the corresponding author upon reasonable request.

## Supplementary material

Supplementary Figure 1 Immunofluorescence shows positive expression of CAPZA1 in wild-type (WT) samples, whereas its expression is reduced in CAPZA1 ^c.11T>C^ mutant samples. Scale bars = 5 μm.

Supplementary Figure 2 Immunofluorescence identification of round spermatids. Immunofluorescence staining of isolated round spermatids showing expression of ACRV1 (green) and Prm1 (red). Nuclei were counterstained with DAPI (blue). *n* = 3. Scale bar = 10 μm.

Supplementary Figure 3 Gene expression analysis of key regulators in mouse round spermatids. RT-qPCR analysis of ATF4 **(A)**, CREBBP **(B)**, EP300 **(C)**, KAT2A **(D)**, KAT2B **(E)**, NFE2L2 **(F)**, SLC3A2 **(G)**, and SLC7A11 **(H)** mRNA expression. Data are presented as mean ± SD (*n* = 3). ^*^*p* < 0.05, ^**^*p* < 0.01, ^***^*p* < 0.001 compared with NC group. “ns” indicates no significant difference between groups.

Supplementary Figure 4 ChIP-qPCR analysis showing enrichment of EP300 **(A)** and H3K27ac **(B)** at the target loci in NC and KO-CAPZA1 round spermatids.

Supplementary Figure 5 Negative controls for immunofluorescence staining. Immunofluorescence images of round spermatids and spermatozoa from normal and KO-CAPZA1 groups stained with secondary antibody only, without primary antibody incubation. No detectable fluorescence signal was observed, confirming the specificity of primary antibody staining in the main figures. DAPI was used for nuclear counterstaining. Scale bars = 100 μm and 20 μm.
