# Supplementary figures and images for: CAPZA1 deficiency disrupts sperm flagellar structure and motility, potentially involving the p300/SLC7A11 pathway
Source: Front Endocrinol (Lausanne). 2026 Mar 4;17:1744836. doi: 10.3389/fendo.2026.1744836 (PMC12995763; doi:10.3389/fendo.2026.1744836)

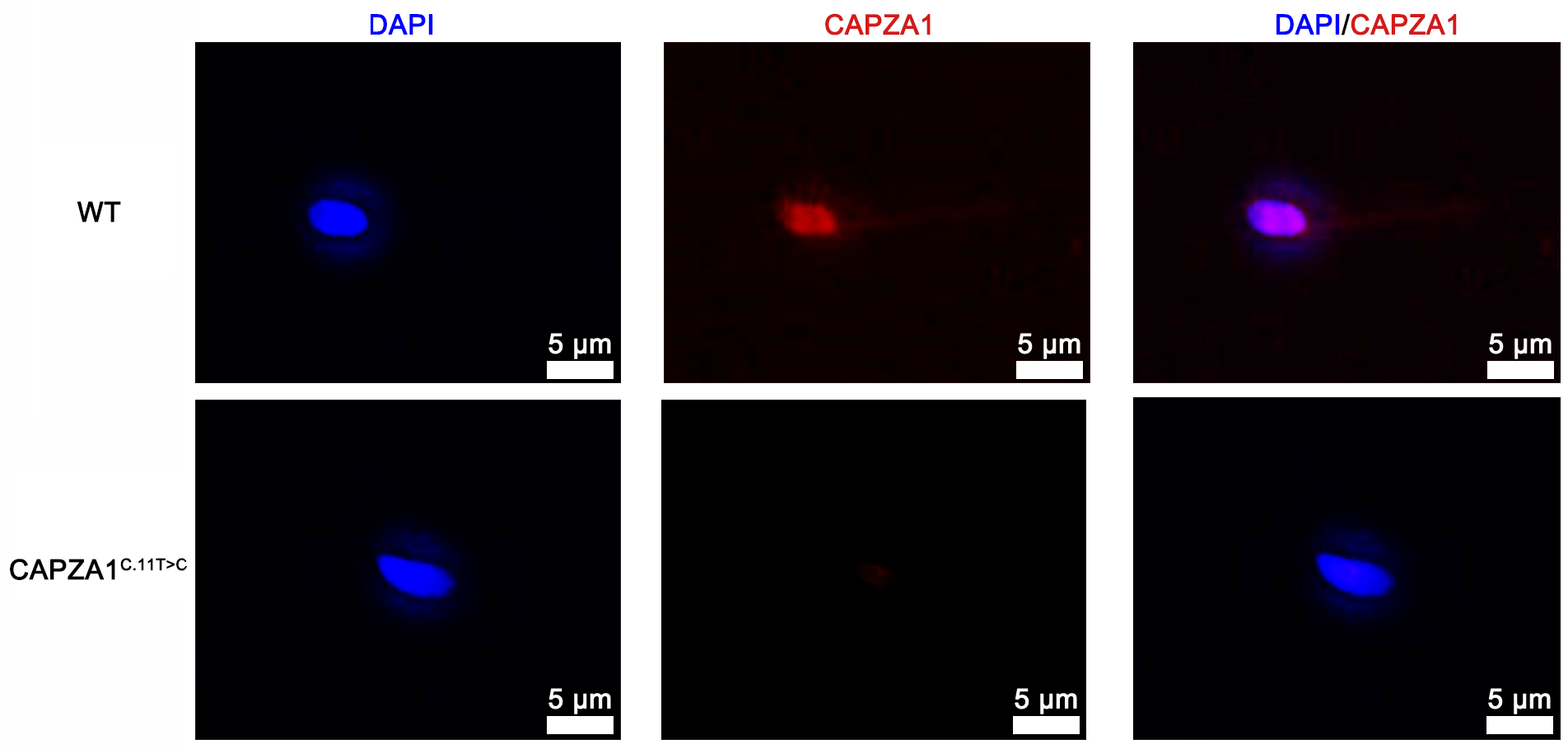

Supplement: Supplementary Figure 1 — Immunofluorescence shows positive expression of CAPZA1 in wild-type (WT) samples, whereas its expression is reduced in CAPZA1 c.11T>C mutant samples. Scale bars = 5 μm. [file Image1.tif]

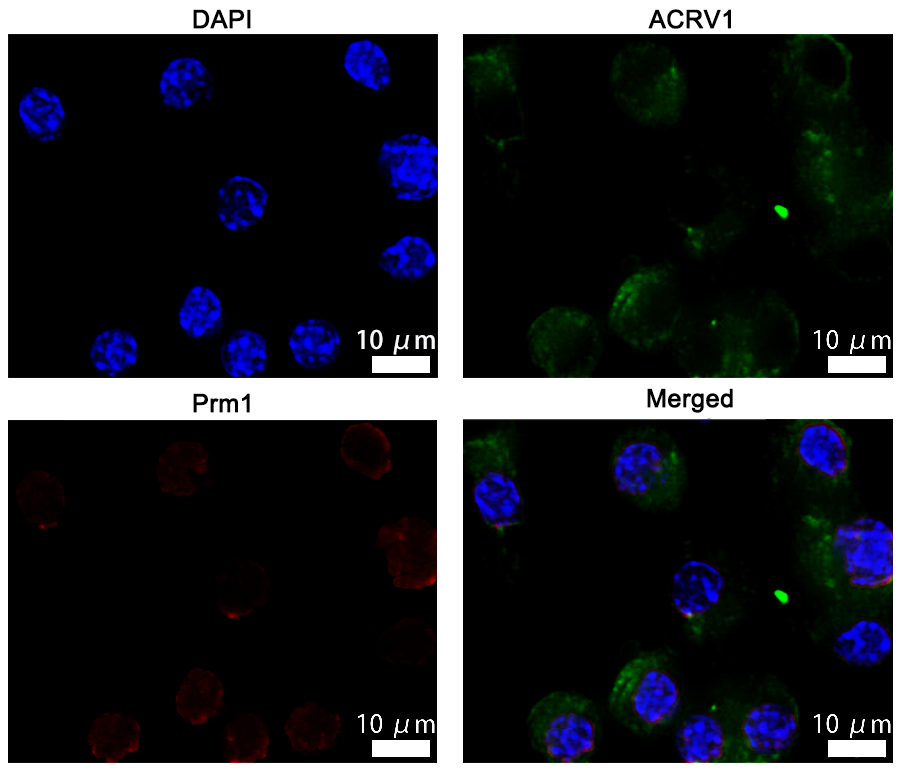

Supplement: Supplementary Figure 2 — Immunofluorescence identification of round spermatids. Immunofluorescence staining of isolated round spermatids showing expression of ACRV1 (green) and Prm1 (red). Nuclei were counterstained with DAPI (blue). n = 3. Scale bar = 10 μm. [file Image2.tif]

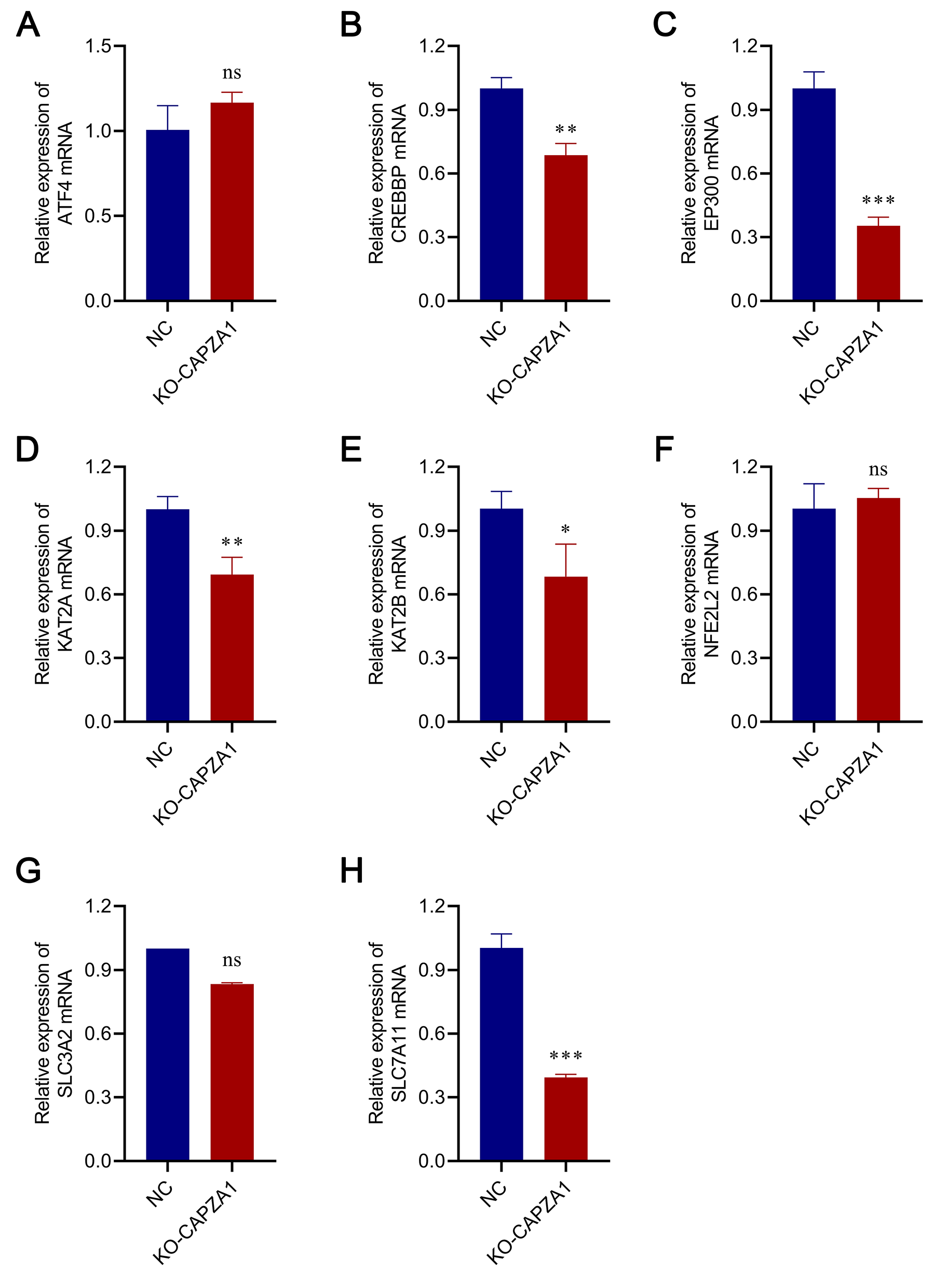

Supplement: Supplementary Figure 3 — Gene expression analysis of key regulators in mouse round spermatids. RT-qPCR analysis of ATF4 (A), CREBBP (B), EP300 (C), KAT2A (D), KAT2B (E), NFE2L2 (F), SLC3A2 (G), and SLC7A11 (H) mRNA expression. Data are presented as mean ± SD (n = 3). *p < 0.05, **p < 0.01, ***p < 0.001 compared with NC group. “ns” indicates no significant difference between groups. [file Image3.tif]

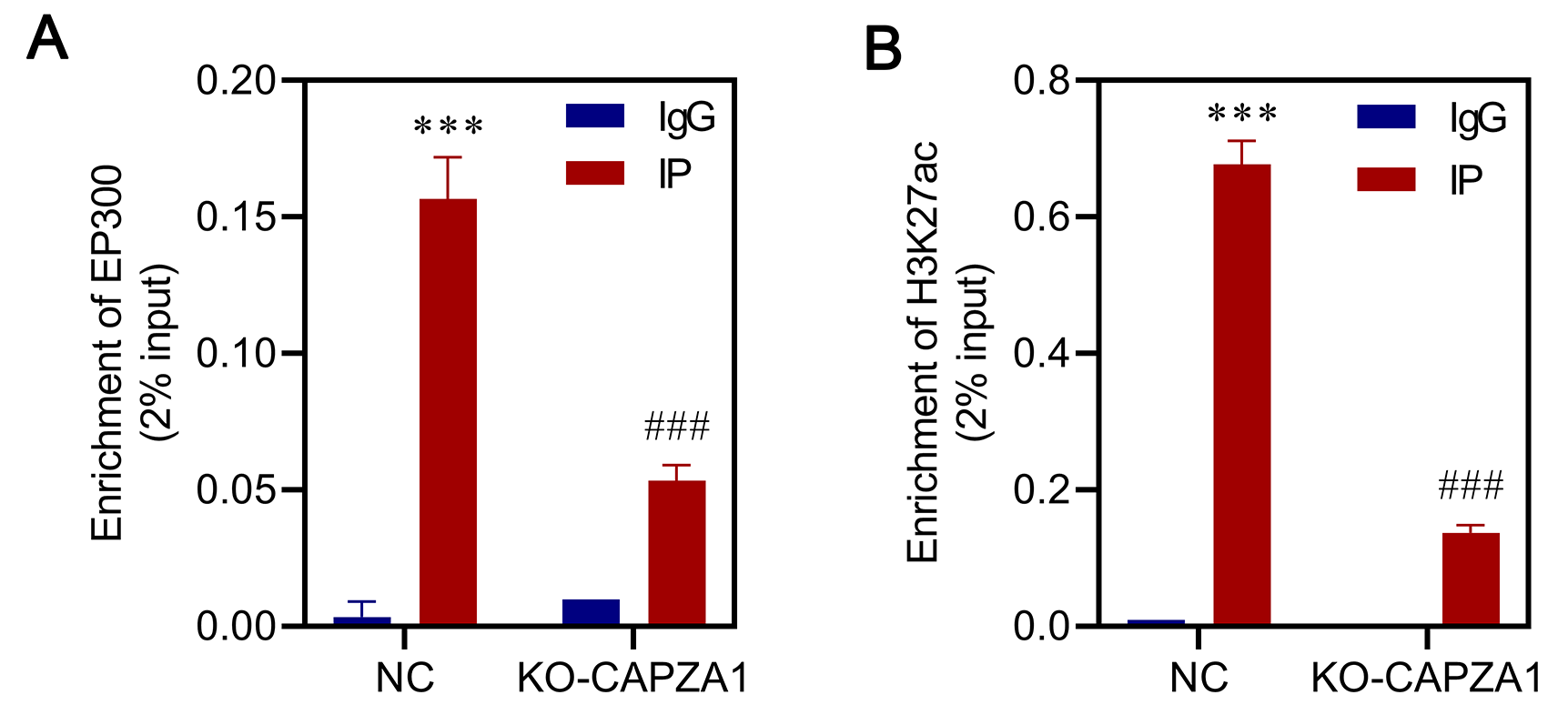

Supplement: Supplementary Figure 4 — ChIP-qPCR analysis showing enrichment of EP300 (A) and H3K27ac (B) at the target loci in NC and KO-CAPZA1 round spermatids. [file Image4.tif]

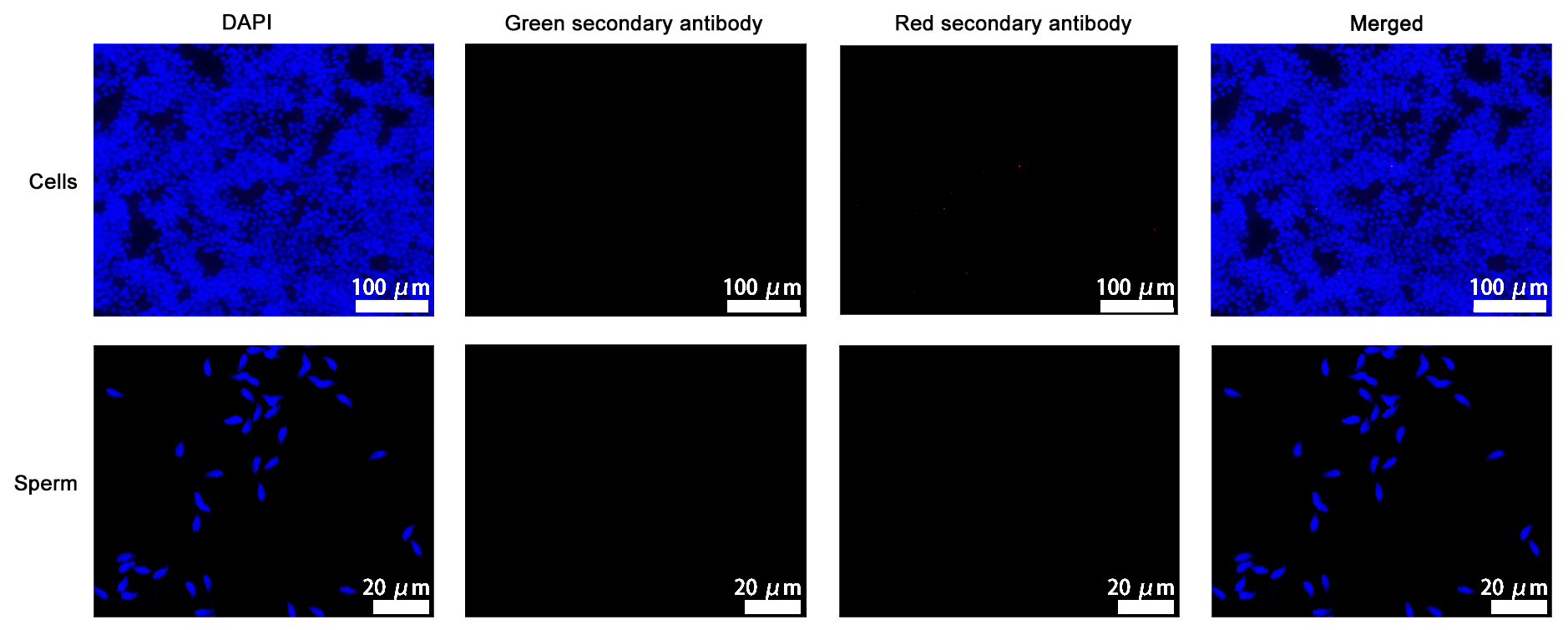

Supplement: Supplementary Figure 5 — Negative controls for immunofluorescence staining. Immunofluorescence images of round spermatids and spermatozoa from normal and KO-CAPZA1 groups stained with secondary antibody only, without primary antibody incubation. No detectable fluorescence signal was observed, confirming the specificity of primary antibody staining in the main figures. DAPI was used for nuclear counterstaining. Scale bars = 100 μm and 20 μm. [file Image5.tif]
