# Supplementary material for: CAPZA1 deficiency disrupts sperm flagellar structure and motility, potentially involving the p300/SLC7A11 pathway
Source: Front Endocrinol (Lausanne). 2026 Mar 4;17:1744836. doi: 10.3389/fendo.2026.1744836 (PMC12995763; doi:10.3389/fendo.2026.1744836)
Supplement: Supplementary file 6 [file Table1.docx]

**Supplementary Table 1. Primer sequences for RT-PCR analysis**

| ***Gene*** | **Primer sequence (Forward)** | **Primer sequence (Reverse)** |
| --- | --- | --- |
| *ATF4* | 5'-AACCTCATGGGTTCTCCAGCGA-3' | 5'-CTCCAACATCCAATCTGTCCCG-3' |
| *CREBBP* | 5'-CACCATCTGTGGCTACTCCTCA-3' | 5'-GGTTTCAGCACTGGTCACAGAG-3' |
| *EP300* | 5'-GTGATGACCCTTCCCAACCTCA-3' | 5'-CTCGTGGTGAAGGACACAGATC-3' |
| *KAT2A* | 5'-CACGGAAATCGTCTTCTGTGCC-3' | 5'-CGTACTCGTCAGCATAGGTGAG-3' |
| *KAT2B* | 5'-CCTCTTCACCTGCGTCCACAAA-3' | 5'-TCTCCAAGGAGCCTTCAACCAC-3' |
| *NFE2L2* | 5'-GCCCTCAGCATGATGGACTT-3' | 5'-AACTTGTACCGCCTCGTCTG-3' |
| *SLC3A2* | 5'-GAGCGTACTGAATCCCTAGTCAC-3' | 5'-GCTGGTAGAGTCGGAGAAGATG-3' |
| *SLC7A11* | 5'-AATACGGAGCCTTCCACGAG-3' | 5'-CTCCAGGGGCAGTCAGTTAG-3' |
| *GAPDH* | 5'-GTGTTCCTACCCCCAATGTGT-3' | 5'-ATTGTCATACCAGGAAATGAGCTT-3' |
